# Epidemiologic Trends of Viral Skin Infections in Egypt: A Cross-Sectional Hospital-Based Study

**DOI:** 10.1155/2019/5469726

**Published:** 2019-03-27

**Authors:** Ramadan Saleh, Essam Nada, Ahmed F. Hamed, Wesam M. Hussien

**Affiliations:** ^1^Department of Dermatology, Venereology and Andrology, Faculty of Medicine, Sohag University, Sohag, Egypt; ^2^Department of Public Health and Community Medicine, Faculty of Medicine, Sohag University, Sohag, Egypt

## Abstract

Viral skin infections (VSIs) were ranked among the top 50 prevalent diseases in 2010. The objective of this study was to determine the epidemiologic features of VSIs in patients attending a dermatology clinic in Egypt from June 2010 to May 2011. Patient's residence, occupation, housing data, and family history of similar conditions were recorded. Categorical data were recorded as frequencies and percentages and were compared by Chi square test. P value < 0.05 was significant. Diagnosis of VSIs was made in 1000/20322 (4.9%) patients. Out of the 1000 patients with VSIs, 580 (58.0%) were residents of rural areas and 420 (42.0%) were residents of urban areas (p = 0.02). Out of the 1000 patients, 489 (48.9%) were females and 511 (51.1%) were males (p = 0.25). The breakdown of 1000 patients with VSIs indicated diagnosis of viral warts in 673 (67.3%), chickenpox (CP) in 200 (20.0%), herpes simplex (HS) facialis in 50 (5.0%), herpes zoster (HZ) in 42 (4.2%), molluscum contagiosum (MC) in 27 (2.7%.0), and anogenital warts in 8 (0.8%) cases. Overcrowding (sharing a bedroom by more than 3 persons) was recorded in 652/1000 (65.2%) of the patients with VSIs [165/200 (82.5.3%) in CP, 36/50 (72%) in HS facials, 427/673 (63.4%) in viral warts, 14/27 (51.9%) in MC, and 10/42 (23.8%) in HZ]. Family history of a similar condition was positive in 329/1000 (32.9%) of the patients with VSIs [142/200 (71.0%) in CP, 177/673 (26.3%) in viral warts, 5/27 (18.5%) in MC, and 4/50 (8%) in HS facialis]. In conclusion, viral warts and CP were the commonest VSIs diagnosed in patients who attended a dermatology clinic in Egypt. Viral skin infections were more prevalent among patients who lived in rural areas and under crowded conditions. These data may have important public health implications particularly in developing countries.

## 1. Introduction

Viral skin infections (VSIs) were ranked among the top 50 prevalent diseases in 2010 with a global prevalence of 122,601,000 [1]. Common VSIs include herpes simplex (HS), herpes zoster (HZ), molluscum contagiosum (MC), anogenital warts, and viral warts [2]. Geographically representative data, on the periodic evaluation of epidemiology of VSIs, are important from clinical and public health perspectives. The objective of this study was to determine the epidemiologic features of VSIs in a cohort of patients attending a dermatology clinic in Egypt.

## 2. Patients and Methods

The study was approved by the Institutional Research and Ethical Committees, Faculty of Medicine, Sohag University, Egypt. This cross-sectional study included patients who attended a dermatology clinic at Sohag University Hospitals, a tertiary care medical facility in Upper Egypt, between June 2010 and May 2011, and were diagnosed as having VSIs. Patients from all age groups and both sexes were included. Patient's residence, occupation, housing data (number of persons per room), and family history of similar conditions were obtained from the participants. Dermatological examination was conducted by a senior dermatology resident and was verified by a dermatology consultant.

The classification and clinical diagnosis of VSIs were made following the tenth edition of the International Statistical Classification of Diseases (ICD-10) [3] and the eighth edition of Rook's Text Book of Dermatology [4], respectively. Accordingly, VSIs were listed as viral warts (verrucae), anogenital (venereal) warts, HS facialis, chickenpox (CP), HZ, and MC [3]. Categorical data were recorded as frequencies and percentages and were compared by Chi square test. Level of significance was considered at p value < 0.05.

## 3. Results

In this study, clinical diagnosis of VSIs was made in 1000/20322 (4.9%) patients who attended a dermatology clinic over a period of one year. Out of the 1000 patients with VSIs, 580 (58.0%) were residents of rural areas and 420 (42.0%) were residents of urban areas (p = 0.02). Out of the 1000 patients, 489 (48.9%) were females and 511 (51.1%) were males (p = 0.25).

The clinical patterns, frequencies, and percentages of VSIs, at different age groups and in both sexes, are shown in Table 1. The breakdown of the 673 patients with viral warts indicated a clinical diagnosis of common warts in 302 (44.9%), plantar warts in 267 (39.8%), plane warts in 38 (5.8%), filiform warts in 17 (2.5%), and mixed varieties (more than one type of viral warts) in 47 patients (7.0%). Out of the 673 patients diagnosed with viral warts, 189 (28.1%) were students, 172 (25.6%) were manual workers, and 106 (15.8%) were housewives.

Family history of a similar condition was positive in 329/1000 (32.9%) of the patients with VSIs [142/200 (71.0%) of the patients with CP, 177/673 (26.3%) of the patients with viral warts, 5/27 (18.5%) of the patients with MC, and 4/50 (8%) of the patients with HS facialis]. Overcrowding (sharing a bedroom by more than 3 persons) was recorded in 652/1000 (65.2%) of the patients with VSIs [165/200 (82.5.3%) of the cases with CP, 36/50 (72%) of the cases with HS facials, 427/673 (63.4%) of the cases with viral warts, 14/27 (51.9%) of the cases with MC, and 10/42 (23.8%) of the cases with HZ].

## 4. Discussion

Viral skin infections account for a significant portion of dermatologic diseases, often resulting in, or as a consequence of, a disruption in the skin integrity [2]. Previous studies on VSIs were directed towards investigating the epidemiologic and clinical features of selected varieties of viruses such as human papilloma virus [5, 6] or HS virus type 2 [7–9]. Other studies were aimed at examining the role of cutaneous viral infections in the pathogenesis of skin and extracutaneous cancers [10, 11]. However, data on the global epidemiologic aspects of VSIs are lacking. In this study, VSIs were diagnosed in 4.9% of all patients who attended an outpatient dermatology clinic in Upper Egypt and were more frequently seen among rural residents. In previous epidemiological studies of skin diseases, using hospital-based data, the rate of VSIs was 7.35% in Cairo, Egypt [12], 6.1% in Nigeria [13], 6.4% in Iran [14], and 12.8% in Saudi Arabia [15].

Viral warts were the commonest VSIs, observed in this study, and accounted for approximately 2/3 of all cases. Similarly, viral warts accounted for high proportions of VSIs in previous studies from Cairo, Egypt (75.05%) [12], Nigeria (40.4%) [13], Iran (55.1%) [14], and Saudi Arabia (65.64%) [15]. In this study, common warts and plantar warts were the commonest varieties of viral warts and represented more than 3/4 of all cases. Viral warts were more prevalent among students, manual workers, and housewives and in overcrowded conditions. Increased potential for minor trauma, in manual worker and housewives, and overcrowding may play a role in the transmission of viral warts in those selected categories of patients.

Chickenpox accounted for 1/5 of VSIs, in this study, and was more prevalent among children. Overcrowding and positive family history were recorded in the majority of CP cases included in the current study. Crowded conditions may facilitate direct or indirect transmission of CP among those patients. In previous reports on VSIs, the rate of CP was 6.69% in Cairo, Egypt [12], 10.9% in Iran [14], and 12.8% in Saudi Arabia [15]. Less common types of VSIs, recorded in this study, included HS facialis, HZ, MC, and anogenital warts.

The data emerging from the current study highlighted some epidemiological aspects of VSIs among patients living in Upper Egypt. The rate of VSIs (4.9%), in this study, was lower as compared to the rate of 7.35% reported in a previous national study conducted in Cairo [12]. In both studies, viral warts represented high proportions of VSIs while the rate of CP was lower in the previous study from Cairo (6.69% vs 20.0%). The retrospective nature of the previous study that was conducted in Cairo and inclusion of the majority of patients from urban areas may explain, at least in part, the discrepancies noted in the results of the two studies.

On the other hand, the variation in the epidemiology of VSIs, in this study, from previous international reports [13–15], may be related to factors such as differences in environmental factors, socioeconomic conditions, and changes in the virulence of the cutaneous viral pathogens. Other suggested reasons for disparity in the prevalence of skin diseases globally may include differences in the type of reported prevalence, study methodology, geographic areas, ethnic groups, and age distribution [16]. Continuous and sustainable national and local data collection have been recommended to reduce the burden of disease in developing countries and narrow the health gap between the developed and developing worlds [17].

Despite interesting findings of this study, few limitations were noted. First, the lack of laboratory tests such as polymerase chain reaction or culture precluded us from identification of specific viral species responsible for VSIs. Second, the hospital-based design precluded us from evaluation of the true prevalence of VSIs in the community.

Third, the finding of low rates of genital warts and anogenital herpes viral infections may not be accurate as patients may prefer to attend private clinics or be seen by gynecologists or urologists. Similarly, the rates of CP and MC, recorded in this study, may be underestimated as a considerable proportion of those patients may consult pediatricians rather than dermatologists. Future community-based studies including laboratory tests, for viral identification, may help overcome these shortcomings and provide accurate estimates for the epidemiology of VSIs.

## 5. Conclusion

In this study, viral warts and CP were the commonest varieties of VSIs diagnosed in patients who attended a dermatology clinic in Egypt. Viral skin infections were more prevalent among patients who lived in rural areas and under crowded conditions. These data may have important public health implications particularly in the developing countries. Healthcare strategies directed towards improving the socioenvironmental factors, in those countries, may play a role in the prevention and control of VSIs.

## Figures and Tables

**Table 1:** The clinical patterns, frequencies, and percentages of viral skin infections at different age groups and in both sexes (total number = 1000).

**Clinical pattern**	**Age group**					**Total number (**%**) **
	**<10 years**	**>10-20 years**	**>20-40 years**	**>40-60 years**	**>60 years**	
Viral warts (*∗*B07)	61	164	358	76	14	**673 (67.3)**
Female						**323 (32.3)**
Male						**350 (35.0)**

Chickenpox (*∗*B01)	150	46	4	0	0	**200 (20.0)**
Female						**87 (8.7)**
Male						**113 (11.3)**

Herpes simplex facialis (B0)	15	8	23	4	0	**50 (5.0)**
Female						**37 (3.7)**
Male						**13 (1.3)**

Herpes zoster (*∗*B02)	1	5	3	13	20	**42 (4.2)**
Female						**22 (2.2)**
Male						**20 (2.0)**

Molluscum contagiosum (*∗*B08.1)	11	5	7	4	0	**27 (2.7)**
Female						**19 (1.9**
Male						**8 (0.8)**

Anogenital warts (*∗*A63.0)			7	1		**8 (0.8)**
Female						**1 (0.1)**
Male						**7 (0.7)**

**Total number (**%**)**	**238 **	**228 **	**402 **	** 98 **	** 34 **	**1000 **
	**(23.8)**	**(22.8)**	**(40.2)**	**(9.8)**	**(3.4)**	**(**100%**)**

*∗* means ICD-10: International Statistical Classification of Diseases, version 10.

## Data Availability

The literature review data used to support the findings of this study are included within the article and properly cited.

## References

[1] Hay R. J., Johns N. E., Williams H. C. (2013). The global burden of skin disease in 2010: an analysis of the prevalence and impact of skin conditions. *Journal of Investigative Dermatology*.

[2] Dawson A. L., Dellavalle R. P., Elston D. M. (2012). Infectious skin diseases: a review and needs assessment. *Dermatologic Clinics*.

[3] World Health Organization ICD-10 version 2010 website.

[4] Burns T., Breathnach S., Cox N., Griffiths C. (2010). *Rook’s Textbook of Dermatology*.

[5] Khopkar U. S., Rajagopalan M., Chauhan A. R. (2018). Prevalence and burden related to genital warts in India. *Viral Immunology*.

[6] Chikandiwa A., Kelly H., Sawadogo B. (2018). Prevalence, incidence and correlates of low risk HPV infection and anogenital warts in a cohort of women living with HIV in Burkina Faso and South Africa. *PLoS ONE*.

[7] Domercant J. W., Jean Louis F., Hulland E. (2017). Seroprevalence of Herpes Simplex Virus type-2 (HSV-2) among pregnant women who participated in a national HIV surveillance activity in Haiti. *BMC Infectious Diseases*.

[8] Renesme L. (2017). Herpès néonatal : épidémiologie, manifestations cliniques et prise en charge. Recommandations pour la pratique clinique du Collège national des gynécologues obstétriciens français (CNGOF). *Gynécologie Obstétrique Fertilité & Sénologie *.

[9] Nasrallah G. K., Dargham S. R., Mohammed L. I., Abu-Raddad L. J. (2018). Estimating seroprevalence of herpes simplex virus type 1 among different Middle East and North African male populations residing in Qatar. *Journal of Medical Virology*.

[10] Rollison D. E., Schell M. J., Fenske N. A. (2018). Cutaneous Viral Infections Across 2 Anatomic Sites Among a Cohort of Patients Undergoing Skin Cancer Screening. *The Journal of Infectious Diseases*.

[11] Hampras S. S., Locke F. L., Chavez J. C. (2017). Prevalence of cutaneous viral infections in incident cutaneous squamous cell carcinoma detected among chronic lymphocytic leukemia and hematopoietic stem cell transplant patients. *Leukemia & Lymphoma*.

[12] El-Khateeb E. A., Imam A. A., Sallam M. A. (2011). Pattern of skin diseases in Cairo, Egypt. *International Journal of Dermatology*.

[13] Henshaw E. B., Olasode O. A. (2015). Skin diseases in Nigeria: The Calabar experience. *International Journal of Dermatology*.

[14] Baghestani S., Zare S., Mahboobi A.-A. (2005). Skin disease patterns in Hormozgan, Iran. *International Journal of Dermatology*.

[15] Parthasaradhi A., Al Gufai A. F. (1998). The pattern of skin diseases in Hail region, Saudi Arabia. *Annals of Saudi Medicine*.

[16] Andersen L. K., Davis M. D. (2016). Prevalence of Skin and Skin-Related Diseases in the Rochester Epidemiology Project and a Comparison with Other Published Prevalence Studies. *Dermatology*.

[17] Defo B. K. (2014). Demographic, epidemiological, and health transitions: are they relevant to population health patterns in Africa?. *Global Health Action*.

